# Diffusion histogram analysis predicts progression-free survival in contrast enhancing recurrent IDH mutant gliomas treated with bevacizumab

**DOI:** 10.1007/s11060-026-05782-2

**Published:** 2026-09-09

**Authors:** Elizabeth Loxterkamp, Audrey Luo, Francesco Sanvito, Collin T. Le, Catalina Raymond, Chuyin Yang, Terry J. Prins, Addison Fisher, Noriko Salamon, Linda M. Liau, Robert A. Chong, Phioanh L. Nghiemphu, David A. Nathanson, Timothy F. Cloughesy, Albert Lai, Benjamin M. Ellingson

**Affiliations:** 1 UCLA Brain Tumor Imaging Laboratory (BTIL), Department of Radiological Sciences, David Geffen School of Medicine, University of California, Los Angeles, 924 Westwood Blvd, Suite 615, Los Angeles, CA 90024 USA; 2 Department of Neurology, David Geffen School of Medicine, University of California, Los Angeles, Los Angeles, CA USA; 3 Department of Neurosurgery, David Geffen School of Medicine, University of California, Los Angeles, Los Angeles, CA USA; 4 Department of Molecular and Medical Pharmacology, David Geffen School of Medicine, University of California, Los Angeles, Los Angeles, CA USA; 5 Department of Bioengineering, Henry Samueli School of Engineering and Applied Science, University of California, Los Angeles, Los Angeles, CA USA; 6 Department of Psychiatry and Biobehavioral Sciences, David Geffen School of Medicine, University of California, Los Angeles, Los Angeles, CA USA

**Keywords:** IDH mutant, Diffusion histogram analysis, Bevacizumab

## Abstract

**Background:**

Pretreatment apparent diffusion coefficient (ADC) histogram analysis—specifically the lower Gaussian peak mean (ADC-L)—is a predictive biomarker of progression-free survival (PFS) and overall survival (OS) in recurrent IDH-wildtype glioblastoma receiving anti-VEGF treatment including bevacizumab. IDH-mutant gliomas differ biologically from glioblastoma, as they exhibit lower tumor cellularity, longer survival, and distinct sensitivity to a variety of therapies. Whether ADC-L retains predictive utility in IDH-mutant recurrent glioma has not been established.

**Methods:**

In this retrospective, single-center study, sixty patients with IDH-mutant recurrent glioma (41 astrocytoma, 19 oligodendroglioma) with measurable contrast enhancement and available pretreatment diffusion-weighted MRI who received bevacizumab were analyzed. Univariate and multivariate Cox regression and Kaplan-Meier analyses were performed for PFS and OS.

**Results:**

The optimal ADC-L threshold was 1.19 μm^2^/ms. High ADC-L (≥1.19 μm^2^/ms) was associated with significantly longer PFS (median 5.46 vs. 2.76 months; *p* = 0.006) but not OS. In multivariate analysis, low ADC-L (HR = 2.054; *p* = 0.0228) and astrocytoma histology (HR = 2.295; *p* = 0.0158) were independent predictors of shorter PFS when accounting for number of prior recurrences. ADC-L was also significant predictor of PFS in both astrocytoma and oligodendroglioma patients, independently, after accounting for number of recurrences.

**Conclusions:**

Pretreatment ADC-L predicts PFS in IDH-mutant recurrent glioma receiving bevacizumab. Supporting prospective evaluation of ADC-L as a biomarker for bevacizumab patient selection in this population.

**Supplementary Information:**

The online version contains supplementary material available at https://doi.org/10.1007/s11060-026-05782-2.

## Introduction

Gliomas, including glioblastoma, are common primary brain tumors and remain highly refractory to therapy [1]. Mutation of the isocitrate dehydrogenase (IDH) enzymes represent a distinct subtype of adult glioma and are present in >80% of lower-grade (World Health Organization grade 2/3) gliomas and most often occur in adult patients under the age of 50 [2]. While IDH-mutant gliomas exhibit distinct clinical, molecular, and pathological features from IDH-wildtype glioblastoma, including a more favorable prognosis and therapeutic response, their prognosis remains highly variable and median survival time can range from months to decades [3, 4]. Many IDH-mutant gliomas undergo a process of malignant transformation [5, 6], exhibiting some biologic, radiographic, and clinical features similar to IDH-wildtype glioblastoma [7, 8] including overexpression of VEGF [9] and contrast enhancement on post-contrast T1-weighted MR images [10, 11]. As such, high grade IDH-mutant gliomas including WHO grade 3/4 astrocytomas and WHO grade 3 oligodendrogliomas are often treated with bevacizumab, a humanized VEGF-targeting monoclonal antibody, sometime during their disease course. Bevacizumab is widely indicated for a variety of aggressive cancers, including recurrent high-grade IDH-wildtype glioblastoma [12, 13], it has been studied in recurrent IDH-mutant glioma with suspected malignant transformation [14–18] and pediatric low-grade glioma [19–21] with mixed results. Thus, there is an ongoing need for predictive or prognostic biomarkers to help identify patients that may exhibit therapeutic benefit when treated with bevacizumab.

Multiple single-center and multicenter phase II and III trials (>7 trials in > 400 patients) have suggested that diffusion MRI characteristics are a strong, independent predictive imaging biomarker for anti-VEGF therapeutic efficacy in recurrent IDH-wildtype glioblastoma [22–27]. Specifically, patients with tumors exhibiting an elevated apparent diffusion coefficient in the lower peak of a two-compartment histogram model (ADC-L) in contrast enhancing tumor tissue have significantly longer survival compared with patients having lower ADC-L. While these studies have clearly demonstrated the impact of pretreatment tumor diffusion MR characteristics on outcomes in patients with recurrent glioblastoma, it is not clear whether IDH-mutant malignant gliomas will exhibit the same trends.

Several features of IDH-mutant gliomas make it important to evaluate ADC-L independently of the glioblastoma. First, IDH mutations produce the oncometabolite 2-hydroxyglutarate, which drives CpG island hypermethylation (CIMP) and frequent MGMT promoter methylation, resulting in enhanced sensitivity to alkylating chemotherapy and ionizing radiation compared with IDH-wildtype glioblastoma [28]. IDH-mutant gliomas also generally exhibit higher mean ADC values than IDH-wildtype glioblastoma, reflecting their lower cellularity and unique microstructural organization that could potentially mask survival benefits in patients with high ADC-L [29–31]. Furthermore, the phase II TAVAREC trial found no PFS or OS benefit from the addition of bevacizumab to temozolomide in contrast enhancing recurrent WHO grade 2/3 glioma without 1p/19q codeletion [14], suggesting that imaging biomarker-based enrichment may be critical to identify patients even likely to have a modest benefit from antiangiogenic therapy. Additionally, the molecular distinction between IDH-mutant astrocytoma (1p/19q-intact) and oligodendroglioma (1p/19q-codeleted) may affect VEGF dependence and the magnitude of bevacizumab response, motivating histopathology-stratified analyses. To address this unmet need, we conducted a retrospective, single center study in IDH-mutant patients that received bevacizumab after tumor recurrence and emergence of measurable contrast enhancement to test whether diffusion MR measurements of ADC-L, 1p19q co-deletion, and RANO response were predictive of PFS and/or OS.

## Methods

### Patient selection

**Fig. 1:**
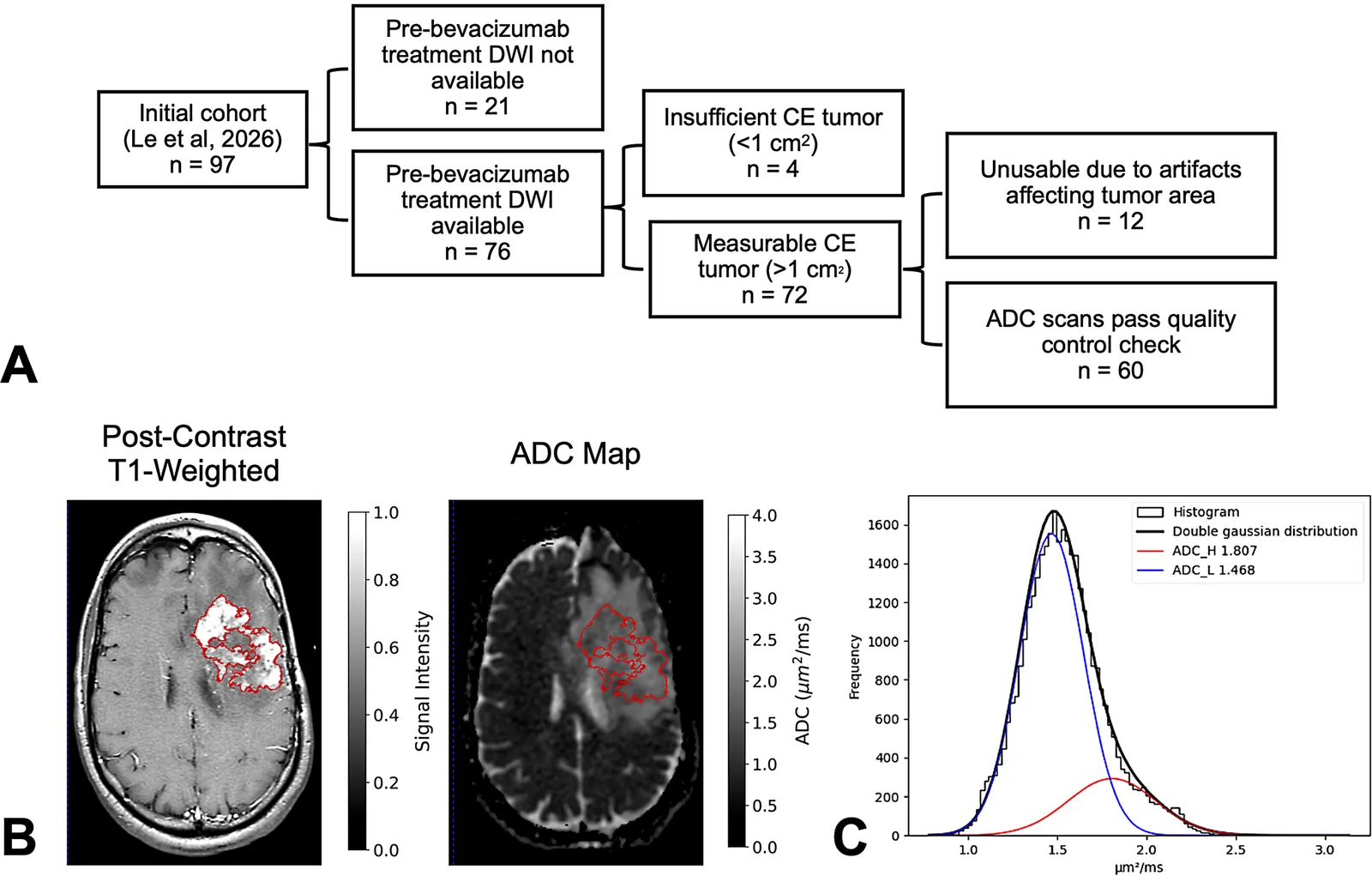
(**A**) Study selection schema. (**B**) Representative post-contrast T1-weighted image, ADC map with tumor outlined in red, and (**C**) ADC histogram of the contrast enhancing tumor

In this UCLA Institutional Review Board-approved study, patients provided informed consent for retrospective access to their clinical and imaging data. This study represents additional diffusion imaging analyses on patients with IDH mutation and either recurrent astrocytoma or oligodendroglioma assessed at UCLA between 1998 and 2024, previously selected and evaluated in Le et al. [32]. Eligibility for the parent cohort included histopathologic diagnosis of glioma, confirmed IDH-mutant status, treatment with bevacizumab at suspected tumor recurrence, and availability of longitudinal MRI scans before bevacizumab initiation. Out of 97 patients meeting the eligibility criteria, 21 of these patients were excluded from the present study due to lack of diffusion-weighted imaging (DWI) scans prior to bevacizumab treatment and 16 of these patients were excluded due to insufficient enhancing tumor size (<1 mL) or distortion/warping on ADC maps in the area of tumor that precluded reliable analysis. Therefore, the final imaging cohort analyzed in this study comprised a total of 60 patients (Fig. 1A). Clinical variables were annotated, including age at bevacizumab initiation, integrated histopathologic and molecular diagnosis [33], MGMT promoter methylation status, concomitant treatments, number of recurrences before bevacizumab initiation, and Karnofsky Performance Score (KPS) at bevacizumab initiation. Progression-free survival (PFS) was calculated based on RANO 2.0 criteria without confirmation scans, as detailed in Le *et al* [32]. In brief, longitudinal measurements of contrast-enhancing tumor volumes were obtained from three-dimensional segmentations, and input into an automated RANO calculator (AIRES) [34]. Progression was defined as *either* ≥ 40% volumetric enlargement of the enhancing tumor compared to baseline or nadir (as flagged by AIRES) *or* as unequivocal non-enhancing progression on T_2_-weighted images (as retrospectively evaluated by a neuroradiologist, blinded to the diffusion data). Unequivocal non-enhancing progression was evaluated qualitatively and defined as a clear extension of the T2/FLAIR hyperintense tumor, with clear features suggesting tumor growth including g mass effect or evidence cortical infiltration. T2/FLAIR alterations ascribable to leukoencephalopathy, vasogenic edema, or macroangiopathic modifications were disregarded. This approach is compliant with one of the RANO 2.0 operational approaches for contrast-enhancing gliomas, consisting in quantitative assessments of the enhancing tumor plus qualitative assessments of the non-enhancing volume [35]. Patients were determined to have achieved RANO radiographic response if, after bevacizumab initiation, the contrast-enhancement (CE) volume of the tumor showed greater than 65% reduction for two scans with ≥4 week separation [35].

### Image acquisition and tumor segmentation

Prior to bevacizumab initiation, pre- and post- contrast T1-weighted, T2-weighted, T2-weighted FLAIR, and DWI scans were acquired. Contrast-enhancing tumor was segmented automatically using the NS-HGlio artificial intelligence tool [36] (Neosoma Inc, Groton, MA, United States, https://neosomainc.com). All patients were determined to have measurable contrast-enhancing disease according to RANO 2.0 criteria. [35] In cases with missing sequences, in order to isolate only contrast-enhancing regions without cystic or necrotic components, the contrast-enhancing tumor component was segmented semi-automatically by manually outlining an approximate tumor region and then applying intensity thresholding on T1-weighted subtraction maps as previously described. [36] Initial quality control and manual edits of segmentations were performed by a trained lab member (CTE), and further quality control in order to ensure alignment between segmentation of contrast enhancing regions with ADC maps was performed by another trained lab member (EL), both under the supervision of a neuroradiologist with 8 years of neuroimaging research experience (FS).

### ADC histogram calculation

As previously described [22, 25, 26], ADC values were extracted from all voxels within the CE tumor volume defined during segmentation (Fig. 1B). ADC histogram analysis was then performed on these values using a double Gaussian mixed model fit via a nonlinear regression. The model was defined as: *p(ADC) = f · N(μ*_*ADC*-*L*_, *σ*_*ADC*-*L*_*) + (1 − f) · N(μ*_*ADC*-*H*_, *σ*_*ADC*-*H*_)

Where *p(ADC)* represents the probability density of ADC values, *f* is the fraction of voxels contributing to the lower distribution, and *N(μ, σ)* denotes a normal (Gaussian) distribution with mean *μ* and standard deviation *σ*. The parameters *μ*_*ADC*-*L*_ and *μ*_*ADC*-*H*_ correspond to the means of the lower and higher Gaussian components, respectively (Fig. 1C). Model fits were manually reviewed for quality control. In cases of poor convergence, nonlinear regression was repeated using alternative initial conditions until stable solutions were achieved. The mean of the lower Gaussian distribution (*μ*_*ADC*-*L*_) was used as the primary imaging biomarker, or ADC-L.

### Statistical analysis

The optimal threshold for ADC-L risk stratification was determined by calculating Mantel-Haenszel hazard ratios and corresponding *p*-values for all potential ADC-L thresholds. Univariate Cox analyses using profile-likelihood significance were performed on clinical and imaging variables of interest, including histopathology, age, tumor volume and percent reduction, MGMT status, reason for bevacizumab initiation, KPS, treatment regimen, ADC-L (continuous and categorical), recurrence number, RANO response, and time since radiation therapy (Sup. Table 1). Multivariate Cox regression was performed using variables that were statistically significant (*p* < 0.05) in the univariate analyses. Because histopathology was a significant univariate variable across all patients (*n* = 60), separate univariate and multivariate analyses were also performed within the astrocytoma (*n* = 41) and oligodendroglioma (*n* = 19) subgroups. Significance was determined using the Log-rank (Mantel-Cox) test for PFS curves and the Gehan-Breslow-Wilcoxon test for OS, to account for the long survival tails that violate the proportional-hazards assumption. Data and statistical analyses were conducted using GraphPad Prism 10.6.1 (GraphPad Software Inc, Boston, MA, United States) or MATLAB R2023b v6 (Mathworks Inc, Natick, MA, United States).

## Results

**Fig. 2:**
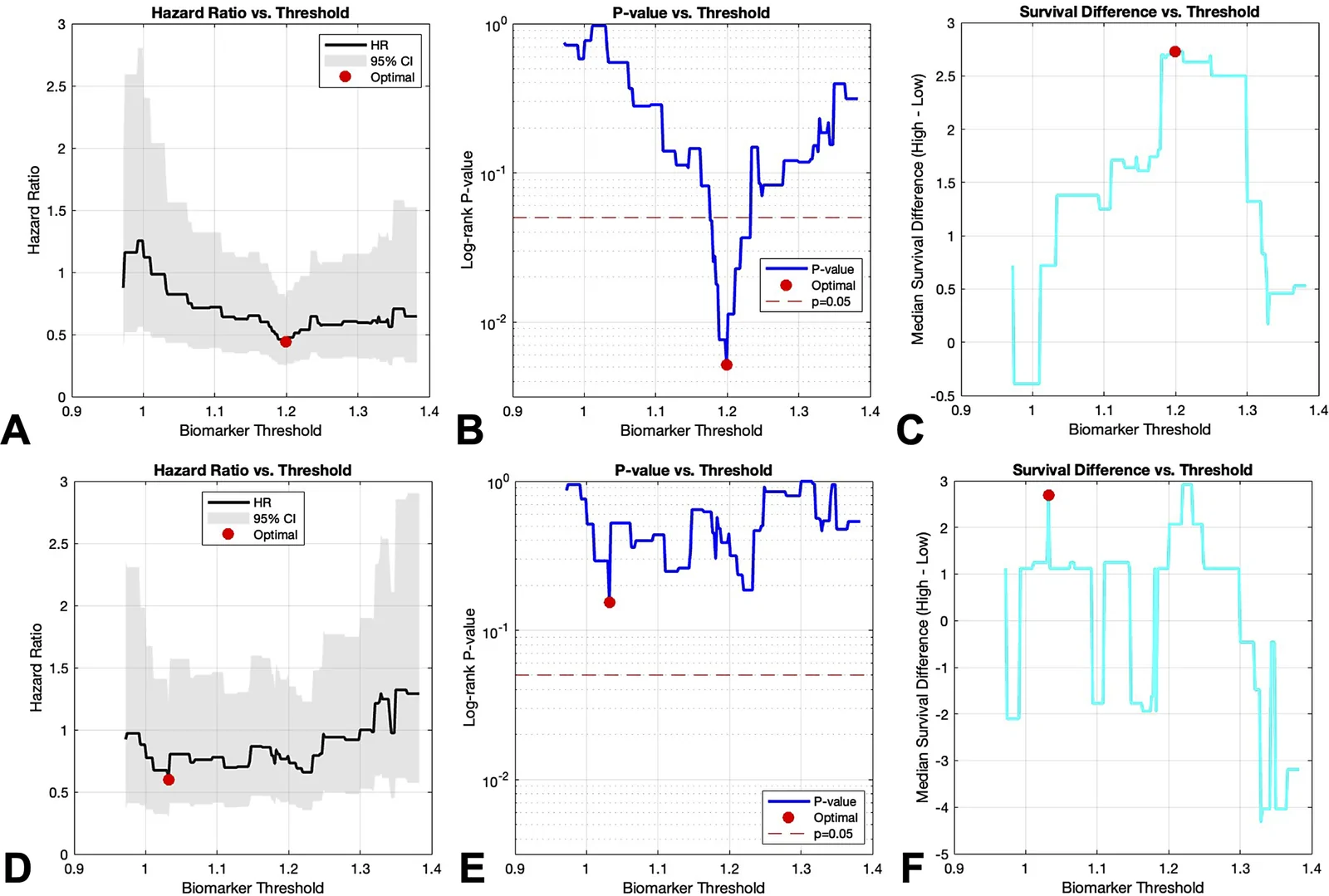
Optimization of ADC-L thresholds for risk stratification. (**A**) Mantel-haenszel hazard ratio (HR) and (**B**) corresponding *p*-value as a function of ADC-L cutoff showing an optimal threshold of 1.19 μm^2^/ms leading to an HR = 0.46 (95% C.I. 0.26 to 0.82) and *p* = 0.0076 for PFS. (**C**) Using 1.19 as the ADC-L threshold yields the highest median survival difference between high and low groups compared to other thresholds. (**D**) Mantel-haenszel hazard ratio (HR) and (**E**) corresponding *p*-value as a function of ADC-L do not yield a cutoff for OS under *p* = 0.05. (**F**) No clear median OS difference between high and low groups across biomarker thresholds

**Fig. 3:**
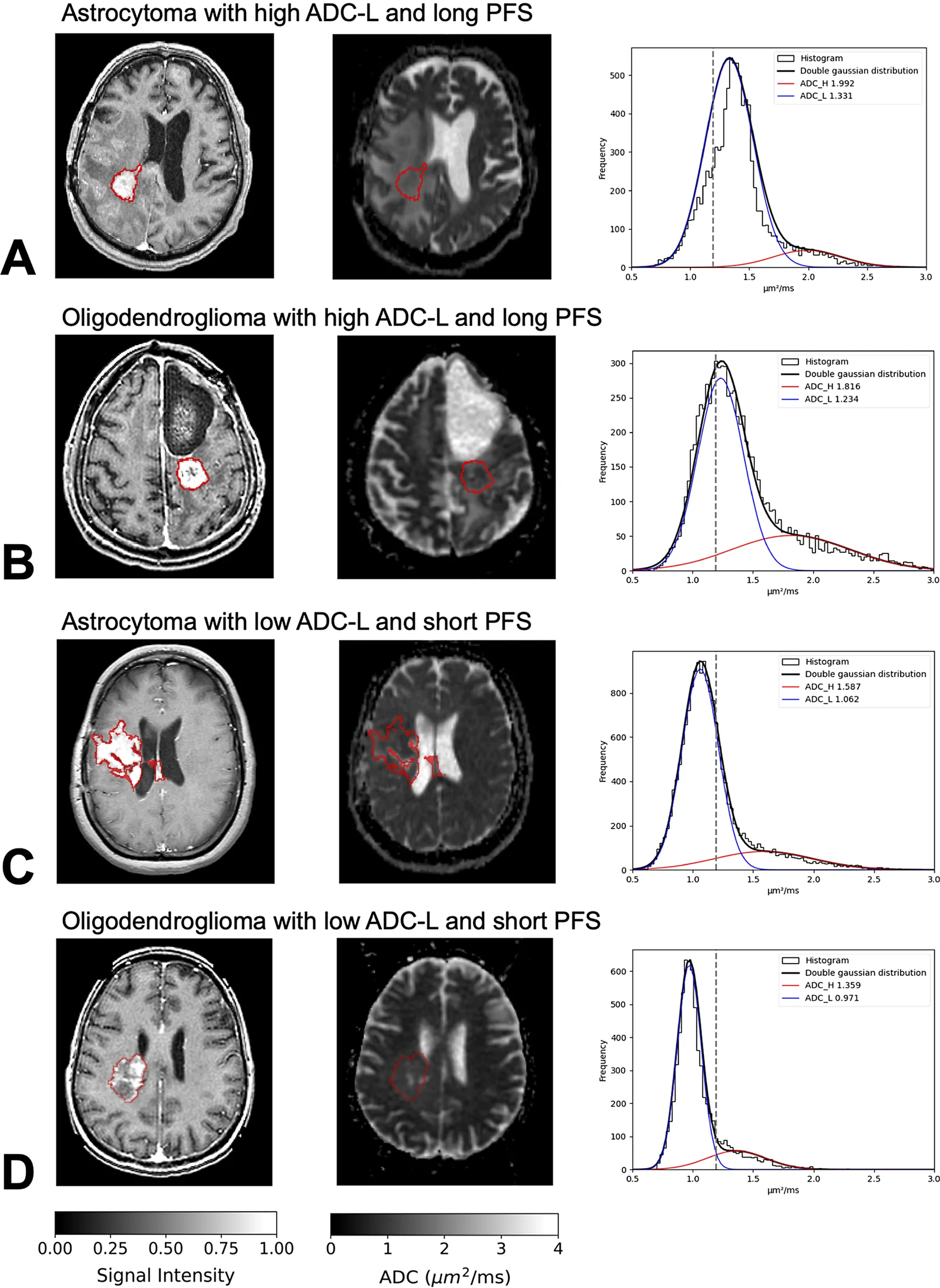
Post-contrast T1-weighted image, enhancing tumor segmentation, ADC colormap, and ADC histogram (left-to-right) in an IDH-mutant (**A**) astrocytoma with high ADC-L and a PFS of 12.8 months; (**B**) oligodendroglioma with high ADC-L and a PFS of 17.4 months; (**C**) astrocytoma with low ADC-L and PFS of 1.4 months; and an (**D**) oligodendroglioma with a low ADC-L and PFS of 1.6 months

All patients meeting eligibility criteria (Fig. 1A) were analyzed for clinical variables including histopathology (astrocytoma, 1p/19q-intact, vs oligodendroglioma, 1p/19q-codeleted), age, CE tumor volume, MGMT methylation status, reason for bevacizumab initiation, KPS, concurrent therapies, recurrence number, timing relative to radiation therapy, and RANO radiographic response (Sup. Table 1). Among the 60 patients, bevacizumab was administered at first (*n* = 8), second (*n* = 13), or third or later (*n* = 39) recurrence. After calculating ADC-L for each patient, the optimal threshold for ADC-L to maximize risk stratification was determined [37]. Results suggested an ADC-L threshold of 1.19 μm^2^/ms resulted in the largest separation in PFS (Fig. 2), which is close to the previously established thresholds for recurrent glioblastoma between 1.2–1.24 μm^2^/ms documented for a variety of anti-VEGF agents [23, 25, 37, 38]. Threshold analysis was also performed on astrocytoma and oligodendroglioma patients separately, but no distinct ADC-L threshold was found in the subgroups (Sup. Fig. 1). Characteristic cases of IDH-mutant patients with high ADC-L and longer PFS demonstrate greater diffusivity in and around the CE area in both astrocytoma and oligodendroglioma patients (Fig. 3A–B) compared to patients with low ADC-L and shorter PFS (Fig. 3C–D).

**Fig. 4:**
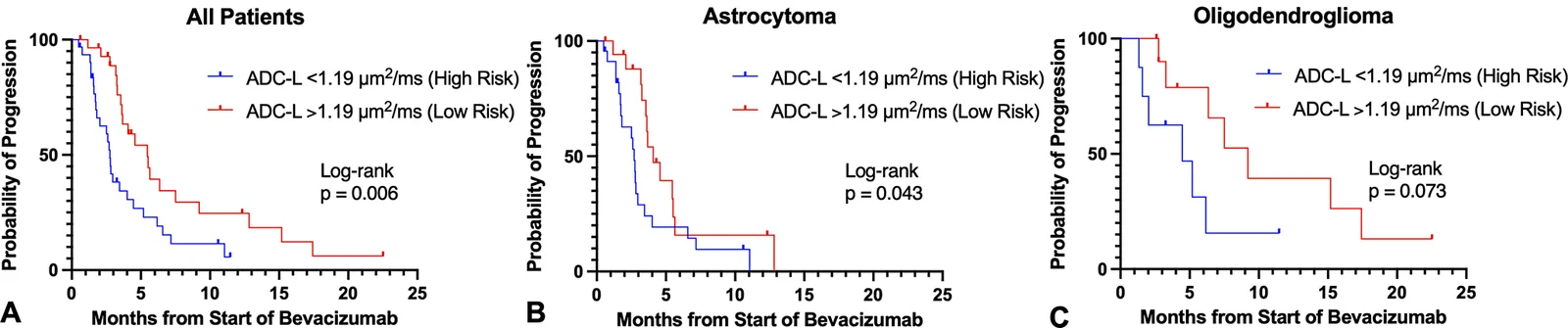
Kaplan-meier plots of PFS stratified by ADC-L > 1.19 μm^2^/ms for (**A**) all patients as well as (**B**) astrocytoma and (**C**) oligodendroglioma, separately. (note that ADC-L significantly stratified PFS after accounting for number of previous recurrences)

Univariate Cox regression analysis (Sup. Table 1) identified ADC-L risk category, tumor histopathology, percent CE volume reduction, and RANO response as significant prognostic factors for PFS. Patients with ADC-L below 1.19 μm^2^/ms had a significantly shorter PFS (Fig. 4A; *p* = 0.0067, HR = 2.3 [95% C.I.=1.3 to 4.4]), with median PFS times of 5.46 months and 2.76 months, respectively. Patients with ADC-L below 1.19 μm^2^/ms had no difference in OS compared with patients exhibiting elevated ADC-L (Sup. Fig. 2A; *p* = 0.38, HR = 1.32 [95% C.I.=0.71 to 2.5]). 1p19q-codeletion (oligodendroglioma) was associated with significantly longer PFS (*p* = 0.010, HR = 0.43 [95% C.I.=0.20 to 0.82]) but not OS (*p* = 0.16, HR = 0.63 [95% C.I.=0.32 to 1.2]). In histopathology-stratified analyses, high ADC-L remained a significant factor for longer PFS in the astrocytoma patients (Fig. 4B; *p* = 0.043), with high ADC-L patients with astrocytoma having median survival of 4.08 months compared to 2.70 months. In oligodendroglioma, high ADC-L was not a significantly associated with longer PFS by (Fig. 4C; *p* = 0.073), though median PFS was more than twice as long (9.21 months and 4.47 months, respectively), suggesting the small subgroup (*n* = 19) may be underpowered. Percent contrast enhancing tumor volume reduction (*p* = 0.015, HR = 1.008 [95% C.I.=1.002 to 1.014 l) and confirmed RANO radiographic response were also significant predictors of PFS (Sup. Table 1; *p* < 0.0001, HR = 0.22 [95% C.I.=0.11 to 0.42]). For OS, contrast enhancing volume reduction was significant only in oligodendroglioma (*p* = 0.014, HR = 1.03 [95% C.I.=1.005 to 1.050]), and RANO response was significant overall (*p* = 0.020, HR = 0.47 [95% C.I.=0.25 to 0.89]) but not within histopathology subgroups. Together, these findings indicate that pretreatment ADC-L, on treatment CE volume reduction, and RANO response after therapy were each associated with longer PFS (Fig. 4A–C; Sup. Table 1).

Multivariable Cox regression was then performed using tumor pathology, ADC-L risk category, and number of previous recurrences as covariates. Results indicated that a diagnosis of astrocytoma (*p* = 0.0158, HR = 2.3 [95% C.I.=1.2 to 4.9]) and ADC-L < 1.19 μm^2^/ms prior to bevacizumab treatment (*p* = 0.0228, HR = 2.1 [95% C.I.=1.1 to 3.9) were significant factors for shorter PFS (Table 1). Because histopathological diagnosis was a significant variable for PFS, separate analyses were run on astrocytoma and oligodendroglioma patients. Results determined that an ADC-L < 1.19 μm^2^/ms was associated with shorter PFS in both patients with astrocytoma (*p* = 0.0478, HR = 2.1 [95% C.I.=1.0 to 4.7]) and oligodendroglioma (*p* = 0.0289, HR = 5.5 [95% C.I. = 1.2 to 31.9]) (Table 1). Because RANO response was a strong predictor of PFS and can only be assessed after initiation of bevacizumab therapy, a landmark survival analysis was performed using the time of RANO determination. In this analysis, the combination of high ADC-L and achievement of RANO radiographic response were significantly associated with improved PFS (Sup. Fig. 3A; *p* = 0.00034) but not OS (Sup. Fig. 3B; *p* = 0.067).

**Table 1:** Multivariable Cox regression analysis

Variable	All (n = 60)				Astrocytoma (n = 41)				Oligodendroglioma (n = 19)			
	PFS		OS		PFS		OS		PFS		OS	
	*p* value	Hazard Ratio (95% C.I.)	*p* value	Hazard Ratio (95% C.I.)	*p* value	Hazard Ratio (95% C.I.)	*p* value	Hazard Ratio (95% C.I.)	*p* value	Hazard Ratio (95% C.I.)	*p* value	Hazard Ratio (95% C.I.)
Tumor histopathology												
Astrocytoma (vs. Oligodendroglioma)	* 0.0158	*2.295 (1.162 to 4.896)*	*+ 0.052*	*1.956 (0.9943 to 4.038)*	–	–	–	–	–	–	–	–
Number of Recurrences												
2nd vs. 1^st^	0.4845	1.44 (0.5234 to 4.331)	0.1558	0.4309 (0.1330 to 1.397)	0.2553	2.093 (0.6040 to 9.646)	0.4471	0.5624 (0.1365 to 2.764)	0.2274	0.1763 (0.005750 to 2.947)	0.6019	0.5044 (0.01985 to 5.996)
3^rd^+ vs. 1^st^	0.1779	1.801 (0.7795 to 4.919)	0.8442	1.093 (0.4765 to 2.960)	0.12	2.402 (0.8150 to 10.25)	0.2985	1.834 (0.6190 to 7.864)	0.7933	0.7936 (0.1535 to 5.759)	0.2408	0.3884 (0.08257 to 2.014)
ADC-L Phenotype												
Low - ADC-L < 1.19 μm^2^/ms (vs. >1.19 μm^2^/ms)	* 0.0228	*2.054 (1.105 to 3.908)*	0.6504	1.158 (0.6141 to 2.214)	* 0.0478	*2.13 (1.007 to 4.692)*	0.9546	0.9782 (0.4610 to 2.141)	* 0.0289	*5.498 (1.192 to 31.85)*	0.4751	1.619 (0.4232 to 6.623)

+ indicates a significance *p* < 0.1, * indicates a significance *p* < 0.05, ** indicates a significance *p* < 0.01, and **** indicates a significance *p* < 0.0001

## Discussion

Consistent with observations in recurrent IDH-wildtype glioblastoma [23–27], the current study confirmed that diffusion MR characteristics of enhancing tumor tissue stratifies outcomes in recurrent IDH-mutant gliomas treated with bevacizumab. Results from the current study suggest that patients with ADC-L higher than 1.19 μm^2^/ms have a significantly longer PFS when compared with patients exhibiting lower ADC-L, even when controlling for clinical factors including histopathological diagnosis. Interestingly, the optimal threshold for risk stratification determined in the current study (1.19 μm^2^/ms) was very similar to previous studies involving recurrent IDH-wildtype glioblastoma (1.2–1.24 μm^2^/ms), suggesting perhaps a common biologic mechanism for this difference in outcome independent of known differences in the underlying tumor biology. Like previous studies in recurrent glioblastoma, all patients in the current study were treated with chemoradiation and had measurable contrast enhancing disease prior to bevacizumab therapy. An increase in diffusivity within tumor tissue after cytotoxic therapy, including radiation and/or alkylating chemotherapies, is well documented in gliomas [39, 40], and a higher measured diffusivity within enhancing disease after radiation therapy has been shown to be associated with a higher risk of post-treatment reactive changes (i.e. pseudoprogression) [41]. While speculative, it is conceivable that ADC-L may effectively stratify patients across different diseases (i.e. IDH-mutant vs. IDH-wildtype) based on the tumor’s reaction to prior therapy, priming the tumor for a better outcome when treated with bevacizumab.

Importantly, the current study detected a strong association between ADC-L and PFS in recurrent IDH-mutant gliomas, but no association between diffusion MR measures and OS as has been demonstrated in IDH-wildtype glioblastoma. Unlike IDH-wildtype glioblastoma, for which no therapy has demonstrated consistent and substantial clinical benefit in the recurrent setting, epigenetic reprogramming in IDH-mutant gliomas enhances sensitivity to a variety of therapies [42], even in the recurrent setting [4]. In this context, PFS is a more relevant endpoint for IDH-mutant gliomas, as it specifically isolates the impact of the experimental treatment under investigation and is not impacted by subsequent therapies that may impact OS [43, 44].

The TAVAREC trial found no PFS or OS benefit from the combination of temozolomide plus bevacizumab in contrast enhancing recurrent WHO grade 2/3 IDH-mutant glioma without 1p/19q codeletion compared with temozolomide alone [14]. However, this trial did not explore diffusion-based differences in patients and also examined the combination of temozolomide and bevacizumab at first recurrence instead of administering bevacizumab as the primary salvage agent. Thus, the present cohort may represent a biologically distinct population more likely to harbor meaningful angiogenic dependence and to benefit from antiangiogenic therapy. These complementary datasets suggest that patient selection using pretreatment ADC-L, combined with the size of contrast enhancing tumor burden, may identify a subgroup of IDH-mutant glioma patients for whom bevacizumab provides meaningful PFS benefit.

Several limitations warrant consideration. This was a retrospective, single-center analysis spanning 26 years (1998–2024), during which MRI scanner hardware, diffusion sequences, and treatment protocols may have evolved considerably. Additionally, ADC measurements, while quantitative and in theory not dependent on traditional sequence parameters like echo time per se, were not harmonized across scanners, potentially introducing variability that attenuated any statistical associations. Additionally, the size of the total cohort in the current study is modest (*N = 60*), and the oligodendroglioma subgroup (*N = 19*) is substantially underpowered for independent subgroup analyses. The ADC-L threshold of 1.19 μm^2^/ms was derived from and evaluated within the same cohort, raising the possibility of overfitting. Despite similarity with the optimal threshold identified in other studies, external validation in an independent IDH-mutant cohort is required before this threshold can be recommended for prospective clinical use. Also, post-progression therapies were not systematically accounted for, limiting the interpretability of OS results. Further, the cohort was restricted to patients with measurable contrast-enhancing disease, likely representing a higher-grade recurrence phenotype enriched for malignant transformation, so findings may not generalize to IDH-mutant gliomas that progress primarily through T2/FLAIR expansion. Lastly, we cannot be certain what fraction of the enhancing volume in any individual patient represented viable tumor as opposed to treatment-related change. No patients underwent re-resection or biopsy at the time of bevacizumab initiation, as tissue is not routinely obtained at the point of transition to antiangiogenic therapy. Since contrast enhancement is a marker of blood-brain barrier disruption, not of tumor per se, disruption arising from recurrent tumor, radiation-induced vascular injury, and from the reactive changes that accompany both are not reliably separable on conventional MRI.

## Conclusion

In summary, the current study establishes pretreatment ADC-L as a significant independent predictor of PFS in IDH-mutant recurrent glioma treated with bevacizumab, extending the predictive utility of this imaging biomarker beyond glioblastoma in which it was originally characterized. The optimal threshold of 1.19 μm^2^/ms closely approximates that established in glioblastoma, suggesting a conserved biological principle across IDH mutation status. These findings support the prospective evaluation of ADC-L as an imaging biomarker for bevacizumab patient selection in IDH-mutant recurrent glioma.

## Electronic supplementary material

Below is the link to the electronic supplementary material.

Supplementary material 1

## Data Availability

All data presented in this paper are available from the authors upon request.
