# Supplementary material for: Diffusion histogram analysis predicts progression-free survival in contrast enhancing recurrent IDH mutant gliomas treated with bevacizumab
Source: J Neurooncol. 2026 Sep 9;179(2):75. doi: 10.1007/s11060-026-05782-2 (PMC13558324; doi:10.1007/s11060-026-05782-2)
Supplement: Supplementary file 1 [file 11060_2026_5782_MOESM1_ESM.docx]

**Supplemental Materials**

**Sup. Table 1. Univariate analysis of clinical variables of interest.**

| Variable | All (n=60) | | | | Astrocytoma (n=41) | | | | Oligodendroglioma (n=19) | | | | |
| --- | --- | --- | --- | --- | --- | --- | --- | --- | --- | --- | --- | --- | --- |
|  | PFS | | OS | | PFS | | OS | | PFS | | OS | | |
|  | *p* value | Hazard Ratio (95% C.I.) | *p* value | Hazard Ratio (95% C.I.) | *p* value | Hazard Ratio (95% C.I.) | *p* value | Hazard Ratio (95% C.I.) | *p* value | Hazard Ratio (95% C.I.) | *p* value | Hazard Ratio (95% C.I.) | |
| Tumor histopathology |  |  |  |  |  |  |  |  |  |  |  |  | |
| Oligodendro- glioma | Ref | Ref | Ref | Ref | -- | -- | -- | -- | -- | -- | -- | -- | |
| Astrocytoma | **** 0.0104*** | ***2.351 (1.213 to 4.922)*** | 0.1649 | 1.582 (0.8324 to 3.174) | -- | -- | -- | -- | -- | -- | -- | -- | |
| Demographics |  |  |  |  |  |  |  |  |  |  |  |  | |
| Age | *+ 0.0579* | *0.9685 (0.9345 to 1.001)* | 0.1444 | 0.9737 (0.9374 to 1.009) | 0.1251 | 0.9719 (0.9331 to 1.007) | 0.3225 | 0.9816 (0.9432 to 1.017) | 0.4724 | 0.9684 (0.8829 to 1.056) | 0.4306 | 0.9618 (0.8663 to 1.057) | |
| Contrast enhancing (CE) volume |  |  |  |  |  |  |  |  |  |  |  |  | |
| Baseline CE volume | 0.4851 | 1.000 (1.000 to 1.000) | 0.4957 | 1.000 (1.000 to 1.000) | 0.5190 | 1.000 (1.000 to 1.000) | 0.9996 | 1.000 (1.000 to 1.000) | 0.4405 | 1.000 (0.9999 to 1.000) | + 0.0922 | 1.000 (1.000 to 1.000) | |
| % CE reduction | **** 0.0145*** | ***1.008 (1.002 to 1.014)*** | 0.1061 | 1.006 (0.9986 to 1.012) | + 0.0730 | 1.007 (0.9993 to 1.013) | 0.5856 | 1.002 (0.9933 to 1.010) | 0.1632 | 1.014 (0.9937 to 1.034) | **** 0.0144*** | ***1.027 (1.005 to 1.050)*** | |
| MGMT promoter |  |  |  |  |  |  |  |  |  |  |  |  | |
| Methylated | Ref | Ref | Ref | Ref | Ref | Ref | Ref | Ref | Ref | Ref | Ref | Ref | |
| Unmethylated | 0.1996 | 1.692 (0.7523 to 3.764) | 0.746 | 1.144 (0.4930 to 2.560) | 0.8143 | 1.118 (0.4474 to 3.020) | 0.9364 | 0.9638 (0.3869 to 2.436) | Not estim-able | 0.5601 | Not estim-able | 0.1400 | |
| Unknown | 0.1705 | 1.630 (0.8109 to 3.359) | 0.1811 | 1.615 (0.7996 to 3.335) | 0.5814 | 1.292 (0.5281 to 3.443) | 0.9359 | 1.038 (0.4155 to 2.632) | 0.2856 | 1.894 (0.5754 to 6.254) | + 0.0506 | 3.195 (0.9970 to 12.07) | |
| Reason for bev initiation |  |  |  |  |  |  |  |  |  |  |  |  | |
| Suspected tumor progression | Ref | Ref | Ref | Ref | Ref | Ref | Ref | Ref | Ref | Ref | Ref | Ref | |
| Worsened edema w/o progression | 0.7288 | 0.8159 (0.1961 to 2.275) | 0.7211 | 1.249 (0.2999 to 3.494) | 0.5602 | 0.6631 (0.1052 to 2.282) | 0.7379 | 0.7833 (0.1231 to 2.752) | 0.8117 | 1.303 (0.06923 to 7.431) | 0.432 | 2.616 (0.1362 to 16.34) | |
| Suspected radiation necrosis | 0.1234 | 0.2802 (0.01571 to 1.313) | 0.9051 | 1.092 (0.1767 to 3.616) | **** 0.0378*** | ***Not estim-able*** | 0.5503 | 0.5687 (0.03156 to 2.761) | 0.9481 | 1.073 (0.05729 to 6.011) | 0.3449 | 3.301 (0.1698 to 21.58) | |
| Steroid tapering w/o progression | 0.7002 | 1.343 (0.2160 to 4.517) | **** 0.0083*** | ***Not estim-able*** | 0.9858 | 0.9869 (0.1575 to 3.382) | **** 0.0037*** | ***Not estim-able*** | N/A | N/A | N/A | N/A | |
| Unknown | 0.4294 | Not estim-able | 0.7312 | 1.449 (0.08098 to 6.888) | 0.3716 | Not estim-able | 0.9712 | 1.038 (0.05764 to 5.045) | N/A | N/A | N/A | N/A | |
| KPS at bev initation |  |  |  |  |  |  |  |  |  |  |  |  | |
| >70 | Ref | Ref | Ref | Ref | Ref | Ref | Ref | Ref | Ref | Ref | Ref | Ref | |
| ≤70 | 0.5494 | 1.226 (0.6419 to 2.511) | 0.3365 | 0.7184 (0.3793 to 1.437) | 0.5306 | 0.7578 (0.3433 to 1.909) | *+ 0.0735* | 0.4458 (0.2012 to 1.087) | 0.3329 | 0.5637 (0.1504 to 1.762) | 0.8526 | 0.8973 (0.2638 to 2.765) | |
| Treatment Scheme |  |  |  |  |  |  |  |  |  |  |  |  | |
| Bevacizumab monotherapy | Ref | Ref | Ref | Ref | Ref | Ref | Ref | Ref | Ref | Ref | Ref | Ref | |
| + Cytotoxic Therapy | 0.4093 | 1.351 (0.6690 to 2.901) | 0.1514 | 1.706 (0.8265 to 3.754) | 0.9972 | 1.002 (0.4095 to 2.804) | 0.4967 | 1.376 (0.5637 to 3.848) | 0.4789 | 1.579 (0.4456 to 6.236) | *+ 0.0689* | Not estim-able | |
| +Immunotherapy | 0.6209 | 1.357 (0.3638 to 4.118) | 0.8581 | 1.111 (0.3073 to 3.260) | **** 0.0270*** | ***5.173 (1.225 to 20.19)*** | 0.6484 | 1.349 (0.3427 to 4.762) | *+ 0.0572* | Not estim-able | 0.7742 | 0.7222 (0.03500 to 5.379) | |
| +IDH Inhibitor | 0.1032 | 2.207 (0.8446 to 5.532) | 0.1579 | 2.291 (0.7029 to 6.572) | 0.3239 | 1.799 (0.5491 to 5.929) | 0.3737 | 1.947 (0.4035 to 7.577) | 0.3233 | 2.547 (0.3396 to 13.90) | 0.2908 | Not estim-able | |
| Recurrence at bev initiation |  |  |  |  |  |  |  |  |  |  |  |  | |
| 1^st^ | Ref | Ref | Ref | Ref | Ref | Ref | Ref | Ref | Ref | Ref | Ref | Ref | |
| 2^nd^ | 0.4276 | 1.507 (0.5520 to 4.490) | 0.276 | 0.5293 (0.1650 to 1.698) | 0.4375 | 1.653 (0.4879 to 7.504) | 0.4483 | 0.5636 (0.1372 to 2.766) | 0.9772 | 0.9653 (0.04429 to 10.33) | 0.8522 | 0.806 (0.03901 to 6.632) | |
| 3^rd^+ | 0.2543 | 1.638 (0.7192 to 4.415) | 0.8786 | 1.071 (0.4753 to 2.863) | 0.1256 | 2.395 (0.8030 to 10.30) | 0.2985 | 1.828 (0.6212 to 7.802) | 0.6985 | 1.345 (0.3413 to 8.896) | 0.3288 | 0.4948 (0.1422 to 2.269) | |
| Less than 3 months since RT |  |  |  |  |  |  |  |  |  |  |  |  | |
| No | Ref | Ref | Ref | Ref | Ref | Ref | Ref | Ref | Ref | Ref | Ref | Ref | |
| Yes | 0.5635 | 1.280 (0.5194 to 2.725) | 0.3133 | 1.514 (0.6507 to 3.114) | 0.845 | 0.9010 (0.2646 to 2.329) | 0.6631 | 1.275 (0.3734 to 3.325) | 0.1693 | 2.890 (0.5975 to 11.26) | 0.2566 | 2.071 (0.5531 to 6.476) | |
| Reached RANO radiographic response |  |  |  |  |  |  |  |  |  |  |  |  | |
| No | Ref | Ref | Ref | Ref | Ref | Ref | Ref | Ref | Ref | Ref | Ref | Ref | |
| Yes | **********  ***< 0.0001*** | ***0.2212 (0.1131 to 0.4179)*** | *******  ***0.0196*** | ***0.4740 (0.2496 to 0.8868)*** | **********  ***<***  ***0.0001*** | ***0.1473 (0.05266 to 0.3551)*** | *+ 0.0748* | *0.5037 (0.2297 to 1.071)* | **** 0.0292*** | ***0.2716 (0.07837 to 0.8740)*** | 0.1942 | 0.4652 (0.1440 to 1.502) |  |
| ADC-L |  |  |  |  |  |  |  |  |  |  |  |  | |
| High | Ref | Ref | Ref | Ref | Ref | Ref | Ref | Ref | Ref | Ref | Ref | Ref | |
| Low | ***** 0.0067*** | ***2.314 (1.261 to 4.359)*** | 0.3754 | 1.32 (0.7142 to 2.473) | **** 0.043*** | ***2.09 (1.023 to 4.452)*** | 0.5831 | 1.23 (0.5894 to 2.648) | *+ 0.0863* | *2.924 (0.8540 to 10.48)* | 0.7105 | 1.234 (0.3915 to 3.782) | |

+ indicates a significance *p* < 0.1, * indicates a significance *p* < 0.05, ** indicates a significance *p* < 0.01, and **** indicates a significance *p* < 0.0001.

**SUPPLEMENTAL FIGURE CAPTIONS**

**Sup. Fig. 1.** No ADC-L threshold found for astrocytoma or oligodendroglioma subgroups. (A) Mantel-Haenszel hazard ratio (HR) and (B) corresponding *p*-value as a function of ADC-L cutoff do not show an optimal for PFS for astrocytoma-only subjects. (C) No clear median OS difference between High and Low groups across biomarker thresholds for PFS for astrocytoma-only subjects. (D-F) No clear median OS difference between High and Low groups across biomarker thresholds for PFS for oligodendroglioma-only subjects.

**Sup. Fig. 2.** Kaplan-Meier plots of OS stratified by ADC-L > 1.19 μm^2^/ms for (A) all patients as well as (B) astrocytoma and (C) oligodendroglioma, separately. (D) Kaplan-Meier plot of PFS stratified by ADC-L > 1.19 μm^2^/ms at baseline and achieving RANO radiographic response.

**Sup. Fig. 3.** Kaplan-Meier plot of landmark analysis after confirmation of RANO for PFS (A) and OS (B) stratified by ADC-L > 1.19 μm^2^/ms at baseline and achieving RANO radiographic response.

**Sup. Fig. 1**

**Sup. Fig. 2**

**Sup. Fig. 3**
